# Prevalence, correlates, and gender disparities related to eating disordered behaviors among health science students and healthcare practitioners in Lebanon: Findings of a national cross sectional study

**DOI:** 10.3389/fnut.2022.956310

**Published:** 2022-07-19

**Authors:** Maha Hoteit, Hala Mohsen, Khlood Bookari, Ghadir Moussa, Najwa Jurdi, Nour Yazbeck

**Affiliations:** ^1^Faculty of Public Health, Lebanese University, Beirut, Lebanon; ^2^PHENOL Research Group (Public HEalth Nutrition prOgram Lebanon), Faculty of Public Health, Lebanese University, Beirut, Lebanon; ^3^Lebanese University Nutrition Surveillance Center (LUNSC), Lebanese Food Drugs and Chemical Administrations, Lebanese University, Beirut, Lebanon; ^4^National Nutrition Committee, Saudi Food and Drug Authority, Riyadh, Saudi Arabia; ^5^Executive Department of Monitoring and Risk Assessment, Food Sector, Saudi Food and Drug Authority, Riyadh, Saudi Arabia; ^6^Department of Clinical Nutrition, Faculty of Applied Medical Sciences, Taibah University, Madina, Saudi Arabia

**Keywords:** eating disorders, health sciences, students, practitioners, Lebanese

## Abstract

**Background:**

The raised prevalence of eating disorders (ED) amongst health science students and health professionals is of mounting concern. This study aims to determine the prevalence and correlates of eating disorders risk amongst a sample of Lebanese health science students and healthcare practitioners of both genders.

**Methods:**

This cross-sectional study enrolled a convenient sample of 1,000 participants (mean age: 23 ± 5.4; females: 74.9%) from faculties of health sciences, clinics, pharmacies, and hospitals. The validated Eating Attitudes Test (EAT-26) was used to screen for eating disorders. Anthropometric data were self-reported by respondents to assess their nutritional status.

**Results:**

The risk of eating disorders was prevalent in 22.5% of participants. Females were at higher risk of ED compared to males *p* = 0.03. Eating disorders risk did not differ between students and practitioners (*p* = 0.3). The highest proportion of high-risk participants were students studying nutrition and practitioners (40.9%), outracing their counterparts in nursing (18.7%), medicine (17.8%), pharmacy (17.7%), and midwifery (4.9%) sciences (*p* = 0.02). Most high-risk participants had normal body weight (60.4%), and 28.9% were overweight (*p* = 0.001). Female gender, nutrition profession, and dieting were associated with increasing the odd of ED. Particularly, dieting increased the risk around five times. Further, each 3 participants over 10 were facing binge eating behavior.

**Conclusion:**

This study uncovers an undervalued profession-related-health-disorder in Lebanese health science students and healthcare practitioners. Specific attention should be given to EDs in professional educational programmes across healthcare disciplines.

## Introduction

Eating disorders (ED), defined by the American Psychiatric Association, are severe and persistent disturbances in eating behaviors accompanied by distressing thoughts and emotions (1). Eating disorders have a detrimental impact on psychological and social functioning and physical health (1). In general, anorexia nervosa (AN), bulimia nervosa (BN), and binge eating disorders (BED) are the three main common types of eating disorders (1). Added to these are the avoidant restrictive food intake disorder, pica, rumination disorders, and other specified feeding disorders (1). ED affects up to 5% of the population, and the mean age of onset for AN and BN is between 15 and 19 years old (1). A systematic review of data from 2000 to 2018 (2) found that eating disorders are highly prevalent worldwide, with the most emphasis on females. The estimated female-male ratio for lifetime prevalence of any eating disorder is 4.2 (2). The prevalence of eating disorders had increased from 3.5% in the 2000–2006 period to 7.8% in the 2013–2018 period (2). A recent review of studies in the Arab world found that the prevalence of eating disorders varies between 2 and 54.8%, with a higher risk of binge eating among Kuwaiti and Egyptian Arabs (3). There is no single stimulator leading to eating disorders, and the exact etiology is still not well-defined. However, the scientific consensus is that eating disorders have genetic predispositions that could be worsened by triggering environmental factors, including cultural idealization, mass media, peer pressure, and dieting (4). Virtually, nothing is known about the individual causal processes involved, or how they interact and vary during the disorder's development and maintenance (5). Individuals with eating disorders have the highest mortality rates among other psychiatric diseases (6). AN is associated with a high risk of death, and 10% of anorexic patients are estimated to die within 10 years of diagnosis (2). To add to the burden, the diagnosis of eating disorders is evasive, and many cases go undetected (7). The published data shows differences in the prevalence of eating disorders between diverse demographics, with some groups appearing to be more susceptible than others. Body image dissatisfaction and disordered eating behaviors increase during college age, and health sciences students, in particular, are an endangered subpopulation for eating disorders (8). The latter issue could endure or commence after they've started practicing their profession, with evident data on the topic of disordered eating among healthcare professionals. One study on nutrition students and dietetic professionals identified high predispositions for food restrictions and weight control issues (9). Thinness idealization, relationship changes with food and body associated with nutrition education, and keeping a continuum all contributed to disordered eating amongst nutrition students and practitioners (9). Another study on female nurses found that work stress and pressing shift hours had triggered poor eating habits, manifested by excessive snacking and binging (10). Nighttime shift duty was positively associated with restrained eating and emotional eating among nurses (10). In addition, in multiple preliminary investigations, medical students reported an appreciable prevalence of eating disorders and disordered eating behaviors, including that conducted in Lebanon (11), Egypt (12), Brazil (13), and Pakistan (14). Further, body image dissatisfaction had been reported as an important concern for pharmacy students, which was considered a serious precursor for disordered eating among them (15). As far as we know, at the moment we are drafting this paper, no studies have addressed the eating disorders topic among both students and practitioners enrolled in health professions. Based on preliminary research efforts and the significance of the topic, it becomes critical to provide national data regarding this topic. This data could serve as a foundation for future intervention and awareness programs addressing the eating disorders burden in the target population. Thus, we designed and conducted this study to be the first aiming to determine the prevalence and correlates of eating disorders amongst a sample of Lebanese health science students and healthcare practitioners of both genders.

## Materials and methods

### Study design and recruitment of study participants

The current investigation is a descriptive cross-sectional study conducted over 5 months, from November 2018 to March 2019, enrolling a convenient sample of health science students and healthcare practitioners from health sciences faculties, pharmacies, clinics, and hospitals in the Beirut and Mount Lebanon districts. The research team of this study had approached student participants at the campus of their universities at the faculties of health sciences to ask them to complete the questionnaire in a self-administered manner. Healthcare practitioners were also reached, during the working hours, at their pharmacies, clinics, and hospitals. To be regarded for inclusion, participants had to be between 18 and 64 years old and willing to participate in this study. We further excluded individuals who were diagnosed previously with one or more eating disorders, and inadequately filled out the self-administered questionnaire. The final sample included 603 health science students and 397 healthcare practitioners enrolled in nutrition (32.8%), midwifery (4.1%), medicine (24.6%), pharmacy (18.4%), and nursing (20.1%) health professions.

### Questionnaire

A self-administered questionnaire, composed of 2 main parts, was employed to collect the data between October and June 2018. The first part of the questionnaire assessed demographic and personal information of study participants, including age, gender, position (student or practitioner), health profession, and self-reported body weight and height. Also, this part included a supplementary question assessing participants' dieting status. The second part of the questionnaire was the Eating Attitudes Test (EAT-26) instrument, used to screen for eating disorders among our study participants. EAT-26 includes sections A and B. Section A consists of 26 questions distributed across three subscales: dieting, bulimia and food preoccupation, and oral control. Respondents answered via six-point Likert scales that offered the choices of “always,” “usually,” “often,” “sometimes,” “rarely,” and “never.” Based on the scoring criteria suggested elsewhere (16), a participant may have a total score ranging from 0 to 78. A score of 20 and above is indicative of an eating disorder risk. Section B of the EAT-26 inspected the disordered eating behaviors of participants in the last 6 months, including eating binges, self-induced vomiting, using laxatives, diet pills, or diuretics, exercising more than 60 min, and losing 9 kg (20 pounds) or more of body weight. Respondents had six response options from “never” to “once a day or more.” Disordered eating behaviors were defined as the following: eating binges (at least 2–3 times a month), self-induced vomiting (at least once a month), using laxatives, diet pills, or diuretics (at least once a month), exercising more than 60 min (at least once a day), and losing 9 kg (20 lbs) or more.

### Statistical analysis

All data were analyzed using the Statistical Package of Social Sciences Software (SPSS) (Version 21.0. Armonk, NY: IBM Crop). Data were presented as mean ± *SD* for the continuous variables and as frequencies (*N*) and percentages (%) for the categorical ones. The chi-squared test was used to examine associations between our categorical variables. The binary logistic regression analysis was applied to determine the predictors of eating disorders. A *p*-value lower than 0.05 was considered significant.

### Ethical consideration

The study has been performed based on the ethical standards laid down in the Helsinki Declaration. Ethical approval was obtained from the Ethical Committee at the Lebanese University (#CU-21-18). A consent form was attached to the questionnaire, informing the respondents about their privacy and rights before participating. There was no penalty for not being involved in the study, and the participation was voluntary. Individual responses were confidential as the questionnaire included no identifying information.

## Results

### Demographic characteristics and weight status of study participants

A total of 1,000 participants were included in this study with a mean age of 23 ± 5.4 years old (median age = 22.0 years old). Of them, 74.9% (*n* = 749) were females, and 25.1% (*n* = 251) were males. The sampled population was composed of 603 health sciences students (60.3%) and 397 healthcare practitioners (39.7%). Students studying nutrition and practitioners (32.8%) predominate compared to those in midwifery (4.1%), medicine (24.6%), pharmacy (18.4%), and nursing (20.1%) sciences. Most of our participants (64.7%) were of normal body weight. Also, 21.1% were overweight. Only a few were underweight (7.7%) and obese (6.5%). Besides, about 43% of the participants were dieting (currently or in the past). Female participants (mean ± *SD*: 23 ± 5) were younger than males (mean ± *SD*: 24 ± 7) (*p* = 0.02). Among females, the highest proportion (42.1%) were in the nutrition profession, which was significantly higher than that of their male counterparts (5.2%) (*p* < 0.001). Moreover, females were mostly of normal weight (69.7 vs. males: 49.8%, *p* < 0.001), and near to half (45.1%) of them reported having dieting experiences, *p* = 0.01 (Table 1).

**Table 1:** Demographic characteristics and weight status of study participants.

		**Overall**	**Males**	**Females**	***p-value***
		**(*****n*** = **1,000)**	**(*****n** =* **251)**	**(*****n** =* **749)**	
		***N*** **(%)**	***N*** **(%)**	***N*** **(%)**	
Age in years (Mean ±*SD*)		23 ± 5.4	24 ± 7	23 ± 5	0.02
Position	Student	603 (60.3)	154 (61.4)	449 (59.9)	0.69
	Practitioner	397 (39.7)	97(38.6)	300 (40.1)	
Health profession	Nutrition	328 (32.8)	13 (5.2)	315 (42.1)	< 0.001
	Midwifery	41 (4.1)	0 (0)	41 (5.4)	
	Medicine	246 (24.6)	92 (36.7)	154 (20.5)	
	Pharmacy	184 (18.4)	72 (28.6)	112 (15.0)	
	Nursing	201 (20.1)	74 (29.5)	127 (17.0)	
Weight status	Underweight	77 (7.7)	4 (1.6)	73 (9.7)	< 0.001
	Normal	647 (64.7)	125 (49.8)	522 (69.7)	
	Overweight	211 (21.1)	87 (34.7)	124 (16.6)	
	Obese	65 (6.5)	35 (13.9)	30 (4.0)	
Dieting (currently or in the past)	No	574 (57.4)	163 (64.9)	411 (54.9)	0.01
	Yes	426 (42.6)	88 (35.1)	338 (45.1)	

### Prevalence of eating disorders risk and its correlates

**Figure 1:**
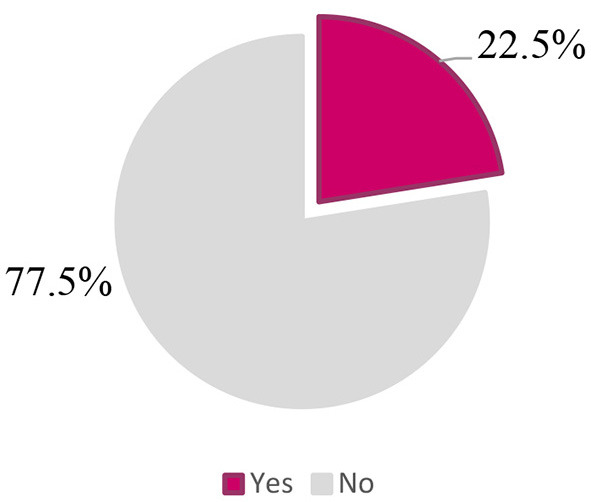
Overall prevalence of eating disorders risk.

**Figure 2:**
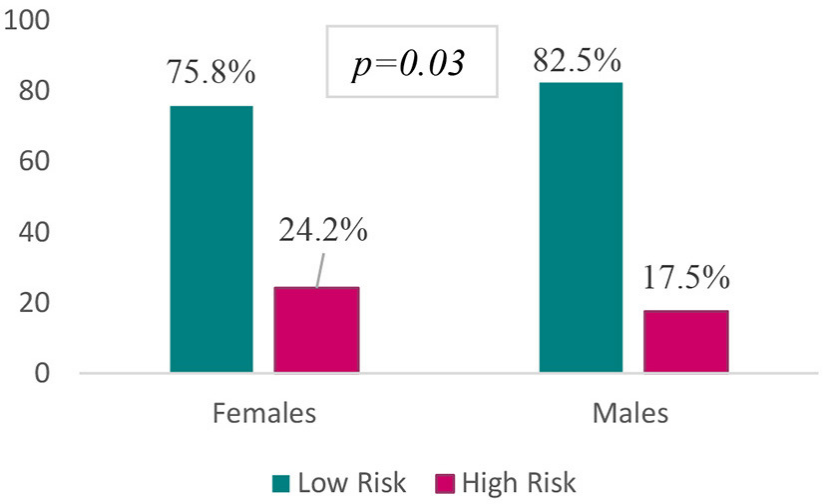
Gender disparities in eating disorders risk.

The overall prevalence of eating disorders risk was 22.5% (Figure 1). Table 2 reveals that the mean age of low-risk (23 ± 5) and high-risk (24 ± 6) participants is just the same (*p* = 0.14), suggesting that participants' age did not contribute to the risk of eating disorders in our sample population. However, participants' gender had a significant association with a possible risk of eating disorders, for which the majority of high-risk participants (80.4%) were females, *p* = 0.03. Furtherly, 24.2% of females were at high risk compared to 17.5% of their male counterparts, *p* = 0.03 (Figure 2). In contrast, participants' position did not appear to predict a higher risk of eating disorders, in which comparable proportions of students (57.3%) and practitioners (42.7%) were susceptible, *p* = 0.30. Further, students studying nutrition and practitioners constituted significantly the predominant proportion (40.9%) of high-risk participants, followed by their counterparts in nursing (18.7%), medicine (17.8%), pharmacy (17.7%), and midwifery (4.9%) sciences, *p* = 0.02. The weight status also appeared to be a significant correlate to participants' risk, and the majority of high-risk participants (60.4%) were of normal body weight, 28.9% were overweight, 4.9% were obese, and 5.8% were underweight, *p* = 0.01. Dieting was a significant associate too; almost three-quarters (72%) of high-risk participants were following a diet, or did so in the past, *p* < 0.001 (Table 2).

**Table 2:** The correlates for the risk of eating disorders.

		**Eating disorders**		***p-value***
		**Low risk**	**High risk**	
		***N*** **(%)**	***N*** **(%)**	
Age in years (Mean ±*SD*)		23 ± 5	24 ± 6	0.14
Gender	Male	207 (26.7)	44 (19.6)	0.03
	Female	568 (73.3)	181 (80.4)	
Position	Student	474 (61.2)	129 (57.3)	0.30
	Practitioner	301 (38.8)	96 (42.7)	
Health profession	Nutrition	236 (30.5)	92 (40.9)	0.02
	Midwifery	30 (3.9)	11 (4.9)	
	Medicine	206 (26.6)	40 (17.8)	
	Pharmacy	144 (18.5)	40 (17.7)	
	Nursing	159 (20.5)	42 (18.7)	
Weight status	Underweight	64 (8.3)	13 (5.8)	0.01
	Normal	511 (65.9)	136 (60.4)
	Overweight	146 (18.8)	65 (28.9)
	Obese	54 (7.0)	11 (4.9)	
Dieting (currently or in the past)	No	511 (65.9)	63 (28.0)	< 0.001
	Yes	264 (34.1)	162 (72.0)	

*Bold values mean significant at p-value < 0.05 for chi-squared test*.

### Predictors of eating disorders among study participants

As the latter findings show that participants' gender, health profession, body weight, and dieting status were associated with the risk of eating disorders among our participants, we further applied the binary logistic regression analysis to specify the predictors of eating disorders. Based on the findings, female participants had a 21% more likelihood of developing eating disorders than males (OR = 1.21, CI = 0.79–1.84, *p* = 0.39). Moreover, students studying nutrition and practitioners had a 21% higher vulnerability for eating disorders in contrast to their counterparts in other health professions (OR = 0.79, CI = 0.56–1.11, *p* = 0.18). However, underweight/normal-weight participants had only 5% higher susceptibility for eating disorders (vs. overweight/obese OR = 0.95, CI = 0.47–1.76, *p* = 0.77). Further, participants who were dieting (currently or in the past) had around 5 times greater risk for eating disorders (vs. non-dieters OR = 4.80, CI = 3.38–6.77, *p* < 0.001) which was the highest among all other predictors (Table 3).

**Table 3:** The predictors of eating disorders.

**Dependent variable: Risk of eating disorders (low risk: reference; high risk)**	**Odds ratio (OR)**	**95% confidence interval (CI)**		***p-value***
		**Minimum**	**Maximum**	
Gender				
Male (Reference)	1	–	–	–
Female	1.21	0.79	1.84	0.39
Health profession				
Nutrition (Reference)	1	–	–	–
Other health professions	0.79	0.56	1.11	0.18
Weight status				
Underweight/Normal (Reference)	1	–	–	–
Overweight/Obese	0.95	0.47	1.76	0.77
Dieting				
No (Reference)	1	–	–	–
Yes	4.80	3.38	6.77	< 0.001

*Bold values mean significant at p < 0.05 for binary logistic analysis test*.

### Disordered eating behaviors among study participants

**Figure 3:**
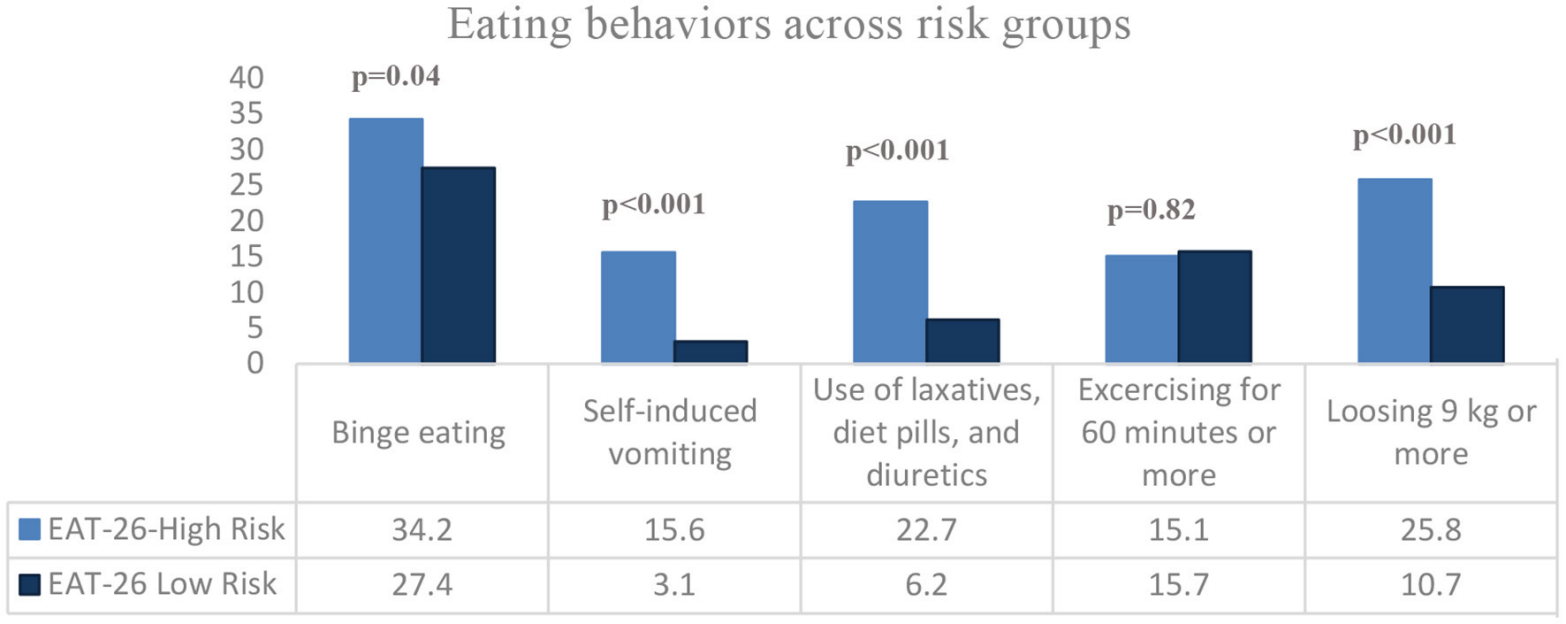
Disordered eating behaviors in study participants.

Figure 3 shows that the highest behavioral ED was binge eating (28.9%), followed by excessive exercise (15.6%), rapid loss of body weight (14.1%), use of laxatives, diet pills, and diuretics (9.9%), and self-induced vomiting (5.9%). The highest proportion of participants who reported behavioral risk for binge eating were significantly females (66.8%, *p* < 0.001), dietitians (28%, *p* = 0.04), and those of normal body weight (58.8%, *p* < 0.001). However, participants' gender (*p* = 0.72 and *p* = 0.21, respectively), work position (*p* = 0.51, *p* = 0.99, respectively), and health profession (*p* = 0.71 and *p* = 0.32, respectively) did not show any statistically significant association with a behavioral risk of self-induced vomiting, and pills use. In contrast, most of the participants having a high risk for self-induced vomiting, and pills use were those having normal body weight (52.5%, *p* = 0.02 and 41.4%, *p* < 0.001, respectively). Moreover, the highest proportion of participants who reported excessive exercise were in the nursing health profession (25.6%) and of normal body weight (59%) (*p* = 0.02, and *p* = 0.01, respectively). Further, the majority who reported rapid weight loss were females (64.5%) and with normal body weight (51.1%), and these associations were statistically significant (*p* = 0.01, and *p* < 0.001, respectively) (Table 4).

**Table 4:** The relationship between participant's characteristics and their behavioral risk.

**In the last 6 months**		**Binge eating**		**Self-induced vomiting**		**Laxatives, diet pills**		**Exercising for more**		**Lose 9 kg or more**						
						**and diuretics use**		**than 60 min**		**of body weight**		***p-values***				
		**Risk**	**No risk**	**Risk**	**No risk**	**Risk**	**No risk**	**Risk**	**No risk**	**Risk**	**No risk**	**(a)**	**(b)**	**(c)**	**(d)**	**(e)**
		***N*** **(%)**	***N*** **(%)**	***N*** **(%)**	***N*** **(%)**	***N*** **(%)**	***N*** **(%)**	***N*** **(%)**	***N*** **(%)**	***N*** **(%)**	***N*** **(%)**
Gender	Males	96 (33.2)	155 (21.8)	18 (27.1)	235 (25)	30 (30.3)	221 (24.6)	42 (26.9)	209 (24.8)	50 (35.5)	201 (23.4)	< 0.001	0.72	0.21	0.57	0.01
	Females	193 (66.8)	556 (78.2)	43 (72.9)	705 (75)	69 (69.7)	679 (75.4)	114 (73.1)	635 (75.2)	91 (64.5)	658 (76.6)					
Position	Students	180 (62.3)	423 (59.5)	38 (64.4)	565 (60.1)	55 (55.6)	548 (60.9)	87 (55.8)	516 (61.1)	87 (61.7)	516 (60.1)	0.41	0.51	0.30	0.21	0.71
	Practitioners	109 (37.7)	288 (40.5)	21 (35.6)	375 (39.9)	44 (44.4)	352 (39.1)	69 (44.2)	328 (38.9)	54 (38.3)	343 (39.9)					
Major	Nutrition	81 (28)	247 (34.7)	18 (30.5)	309 (32.8)	28 (28.2)	299 (33.2)	39 (25)	289 (29.3)	45 (31.9)	283 (32.9)	0.04	0.71	0.32	0.02	0.81
	Midwifery	15 (5.1)	29 (4)	5 (8.4)	39 (4.1)	9 (9)	35 (3.9)	5 (3.2)	39 (4.6)	4 (2.8)	40 (4.6)					
	Medicine	70 (24.2)	176 (24.8)	10 (17.0)	236 (24.8)	19 (19.2)	227 (25.2)	39 (25)	207 (24.5)	39 (27.6)	207 (24.1)					
	Pharmacy	60 (20.7)	124 (17.4)	6 (10.1)	178 (18.9)	20 (20.2)	164 (18.2)	33 (21.1)	151 (17.8)	33 (23.4)	151 (17.6)					
	Nursing	63 (21.8)	138 (19.4)	20 (33.8)	181 (19.8)	23 (23.2)	178 (19.2)	40 (25.6)	161 (19)	20 (14.2)	181 (21)					
Weight status	Underweight	9 (3.1)	68 (9.6)	2 (3.4)	75 (8)	3 (3)	74 (8.2)	5 (3.2)	72 (8.5)	3 (2.1)	74 (8.6)	< 0.001	0.02	< 0.001	0.01	< 0.001
	Normal	170 (58.8)	477 (67.1)	31 (52.5)	615 (65.4)	41 (41.4)	605 (67.2)	92 (59)	555 (65.8)	72 (51.1)	575 (66.9)					
	Overweight	77 (26.6)	134 (18.8)	21 (35.6)	190 (20.2)	39 (39.4)	172 (19.1)	48 (30.8)	163 (19.3)	46 (32.6)	165 (19.2)					
	Obese	33 (11.4)	32 (4.5)	5 (8.5)	60 (6.4)	16 (16.2)	49 (5.4)	11 (7.1)	54 (6.4)	20 (14.2)	45 (5.2)					

*(a) The level of significance related to binge eating*.

*(b) The level of significance related to self-induced vomiting*.

*(c) The level of significance related to laxatives, diet pills, and diuretics use*.

*(d) The level of significance related to exercising for more than 60 min*.

*(e) The level of significance related to losing 9 kg or more of body weight. Bold values mean significant at p < 0.05 for chi-squared test*.

### Eating behaviors across risk groups

**Figure 4:**
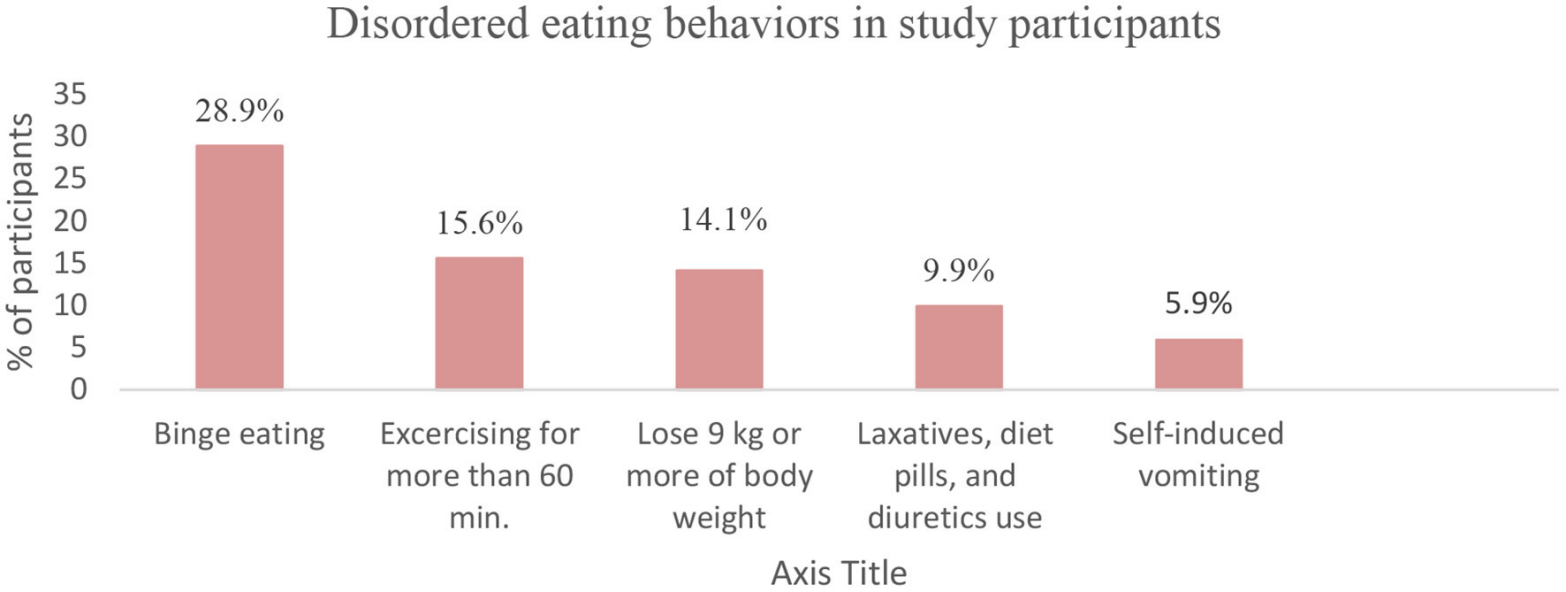
Eating behaviors across risk groups.

Compared to those having low EAT-26 score, high-risk participants significantly reported more disordered behaviors regarding binge eating (34.2 vs. 27.4%, *p* = 0.04), self-induced vomiting (15.6 vs. 3.1%, *p* < 0.001), the use of laxatives, diet pills, and diuretics (22.7 vs. 6.2%, *p* < 0.001), and losing 9 kg or more of body weight (25.8 vs. 10.7%, *p* < 0.001) in the last 6 months. These findings suggest that participants who scored high for EAT-26 scale were more prone to be engaged in different disordered eating behaviors (Figure 4).

## Discussion

In this study, we took the first attempt to provide data about the prevalence of eating disorders and their correlates among a sample of Lebanese health sciences students and healthcare practitioners. Overall, 22.5% of participants appeared to be at high risk for eating disorders, with females being predominant compromising 80.4% of high-risk participants. Female gender, nutrition health profession, and dieting augmented the risk of eating disorders, with dieting being the major contributor by increasing the risk around 5 times. Besides, 28.9% of participants showed a behavioral risk in binge eating, which was the highest proportion among other reported disordered behaviors. Participants with eating disorders risk had more disordered eating behaviors, including binge eating, self-induced vomiting, pills use, and rapid weight loss (9 kg or more) in the last 6 months.

In contrast to preliminary investigations in Lebanon, our obtained prevalence of eating disorders risk is higher than that reported among medical students at the American University of Beirut, in 2017, where 17% of students who completed the EAT-26 scale were at high risk (11). We could relate this disparity in findings to the fact that the current study enrolled both students and practitioners from multiple health professions, including nutrition sciences, who are frequently hypothesized to have a higher prevalence of eating disorders than others. The latter assumption is further confirmed by our study findings, by which nutrition students and practitioners constituted the highest proportion (40.9%) of high-risk participants. In 2007, using the SCOFF questionnaire, a cross-sectional observation of students at the Faculty of Health Care of the Saint Joseph University in Beirut, Lebanon, detected eating disorders among 31.4% of the total population, which exceeds our reported prevalence (17). One plausible explanation for this discrepancy is that the 2007 study was conducted 6 months after the July 2006 war in Lebanon. Wartime is a traumatic life event that may cause an increment in the incidence rates of eating disorders among affected civilians (17).

The obtained prevalence in the current study is lower but comparable to that reported among Saudi female health sciences students (35.4%) (18) and Egyptian medical students in Tanta University (33%) (12). In contrast, a cross-sectional study with 575 medical students in Ain-Shams University found a 12.3% prevalence of eating disorders among Egyptian students, much lower than ours (19). The current findings are in agreement with that reported in Pakistan among a sample of medical students where 23% appeared to have a high risk of eating disorders based on EAT-26 instrument (20). On the other hand, the prevalence of eating disorders among 1,493 college students of the University of Rouen-Normandy, France, exceeded our obtained one only slightly (24.8%) (21). In the United States of America, the prevalence of eating disorder risk among a sample of registered dietitian nutritionists was about 13%, which was less alarming than that obtained in the present study (22). Besides, eating disorders were detected in 36% of the general Navy nurse community (23). Added to these, 8.1% of Saudi female nurses were shown to have eating disorders, including bulimia nervosa and binge eating (24). The understanding of eating disorders was also an interesting debate in some research studies. In one study attempting to assess the eating disorders-related knowledge of pharmacy students, it was evident that most of the participants lacked knowledge, which was also illustrated in other studies amongst healthcare professions (25). Thus, all published data disclosed that eating disorders among health sciences students and healthcare practitioners are of mounting concern in the Arab region and worldwide. However, the prevalence of eating disorders varies either slightly or widely from one country to another. We suggest that the prevalence variations are due to the differences in the study instruments used to screen or assess eating disorders which may cause heterogeneity in findings, the population diverseness, and the cultural background, which may directly impact the risk of eating disorders of demographics.

As also observed in our study, the risk of eating disorders varies significantly according to participants ‘gender, and this is manifested by the finding that females predominate in the high-risk category, having a 21% higher likelihood for the risk of eating disorders than males. The latter finding appears to be in confirmation with a recent study in Lebanon, which showed that 72.7% of female medical students had high risk in the EAT-26 scale (11). Another cross-sectional study with Pakistani medical students provided similar results by observing that 87.9% of high-risk participants were females (14). Also consistent with these findings were that obtained in France (21), the United States and Canada (26), Tunis (27), and Saudi Arabia (18), which all explored that female participants were more vulnerable to eating disorders risk than male participants. The National Comorbidity Survey Replication (NCS-R), a national community household survey of the prevalence and correlates of mental disorders in the United States, found that women have 1.75–3 times higher lifetime prevalence for anorexia, bulimia, or binge eating disorder than men (28). The high vulnerability of females for eating disorders might be related to multiple interlinked factors highlighted in previous research observations. A cross-sectional study on the gender differences in food choice found that women frequently reported avoiding high-fat foods and sugary items (29). Besides, females expressed more perceived unhealthiness for sugars, saturated fats, red meat, white flour, and dairy products. Above these, females reported feeling anxious about having unhealthy meals (29). Not only the types of food do matter, but also females expressed guilty and shameful feelings when eating at specific times of the day. In a study analyzing how frequently and under what circumstances college women did experience feelings of guilt about food, the data indicated that the most “guilty moments” were for snack time, after dinner eating, and in the evening (30). The prevalence of body image dissatisfaction among females is evident in the literature. In line with our findings, in Germany, a cross-sectional study (*N* = 1,338) by Quittkat et al. (31) found that women reported non-satisfaction with their body shape and appearance more than males, which is associated with low self-esteem, disordered eating, and poor health outcomes. Further, women with eating disorders often wish to achieve a BMI of 15–16 kg/m2; however, those without eating disorders are satisfied with a BMI of 18–19 kg/m2 (32). An interview with women who recovered from anorexia nervosa was performed to understand their feelings and perceptions toward their eating behaviors; they reported feeling happy and comfortable when controlling their food intake (33). However, guilt, anguish, sadness, fear, and anger were often associated with eating (33). “Food is considered a villain” was also claimed in the interview, highlighting the negative feelings anorexia nervosa patients usually have (33).

Along with this, the present study showed that more than half of participants who reported binge eating behaviors (66.8%) in the last 6 months were females. A research study in Norway (*n* = 1,846) provided information in this respect, showing that the prevalence of binge eating disorders in women is twice that of bulimia nervosa and five times the prevalence of anorexia nervosa disorders (34). Also of importance, dieting was presented as a major risk factor for the pathogenesis of binge eating (35). An early study showed that women who went on one or more diets within the previous year or engaged in restrained eating behavior showed frequent binge eating episodes (35). In particular, craving for sweets is the prime trigger for binge eating (35). Thus, one reasonable explanation for observing high reports of binge eating among our female participants is that near to half (45.1%) of them reported having dieting experiences.

Also of concern of the current study is that students and practitioners in the nutrition profession constitute significantly the highest proportion (40.9%) of high-risk individuals, with a 21% higher risk when compared with their counterparts in other health professions, including midwifery, medicine, pharmacy, and nursing. Besides, most of participants who had behavioral risk for binge eating were significantly in the nutrition health profession (28%). The latter finding was hypothesized and not unusual due to the abundant data on the burden of eating disorders among this susceptible subpopulation. Our findings are consistent with that of preceding review on the prevalence of eating disorders among nutrition students and dietetic professionals which classified them as being highly prone to experiencing food restrictions and weight control (9). After a cross-sectional comparison with other health professions, German nutrition students showed higher levels of dietary restraint than others, with a particular risk of orthorexia, defined as a stressful focus on eating healthily (36). Moreover, 10% of American nutrition college students were at a high level of concern for developing eating disorders, with 10.3% of them met criteria for food addiction (37). In 2012, an international study (38) found that 77% of nutrition students recognized eating disorders as a concern among their peers. Besides, a pre-enrolment study of students' motivations, awareness, and expectations relating to careers in nutrition and dietetics explored that personal experiences with family or friends, obesity, and experiencing eating disorders were the top motivators to choose nutrition profession (39).

Elaborating more on this topic, 4.6% of 283 surveyed dietitians reported feeling guilty with self-hatred when straying from their diet (40). In addition, 5% of the dietitians admitted avoiding eating away from home or in a social event with others (40). Otherwise, when inevitable, they took their food along when eating away from home (1.1%) (40). Thinness's idealization and implications of food and body associated with nutrition education exposed those in the nutrition profession to higher eating disorder risk, as reported by a recent review (9). The review adds that participants reported that their body shape defines their success as dietitians and that a successful dietitian must have the perfect body shape to convince others (9).

Another interesting finding of the present study is that nursing students and practitioners constituted 18.7% of high-risk participants, which is also an appreciable proportion and could be comparable to that reported by their counterparts in the nutrition profession. In previous investigations, in Turkey, 84.5% of nursing students were at high risk of eating disorders according to EAT-40, and 45.3% were at the risk of developing Orthorexia Nervosa (ON), which were seriously alarming findings (41). The risk of eating disorders among nurses may be related to the high-stress environment they are obliged to adapt to. Work stress and shift duty had been proven to be related to inadequate eating habits among female nurses working in Central Saudi Arabia (10). A high percentage of the female nurses reported eating more fast food, snacks, and binging (10). Further, working nighttime shift duty was positively associated with restrained eating (OR = 1.53; *p* = 0.029) and emotional eating (OR = 1.24; *p* = 0.001) among them (10).

In addition, it is worth to mention that medical students and physicians in our study represented a salient proportion of high-risk participants (17.8%). Medical students had been shown to have a considerable risk of eating disorders in many preliminary investigations, including that reported in Lebanon (11), Egypt (12), Brazil (13), and Pakistan (14). Of note, in this study, the prevalence of eating disorders in the pharmacy health profession was also just the same (17.7%) compared to medicine. A cross-sectional study in Romania found that more than one-third of pharmacy students keep diets to reduce their weight, with participants with high body dissatisfaction tended to have fewer main meals, and to skip breakfasts and dinners (42). Besides, pharmacy students were not satisfied with their body weight, despite not being overweight or obese (15). On the other hand, midwifery was the least profession associated with eating disorders risk, although the risk must not be neglected. Due to the scarcity of data regarding the prevalence and the correlation of eating disorders among midwives and pharmacists, our current data is an initiative to highlight an existing problem that may follow an upward trend in coming observations. Health sciences students and health care practitioners are shown in the current study to have a varied risk for eating disorders, but the problem is still of concern among all professions and highlight the need for further assessment and diagnosis.

An unusual finding is that the predominant proportion of high-risk participants was of normal body weight (60.4%), with overweight participants coming next (28.9%). However, a few of the high-risk participants were obese (4.9%), *p* = 0.01. Besides, normal-weight participants reported significantly the highest risk for binge eating, self-induced vomiting, pills use, excessive exercise, and rapid weight loss in the last 6 months as opposed to underweight, overweight, and obese participants. The latter findings contradict that reported in Russia, which found that bulimia was associated with a higher risk of obesity (OR = 1.03, 95% CI: 1.02–1.05) among 13,341 adults who completed the EAT-26 (43). A systematic review (44) on body image dissatisfaction found that obese individuals had higher dissatisfaction rates than normal-weight individuals, which also comes in incongruence with our findings. Added to these, a study on prevalence and correlates of binge eating disorders in the World Health Organization World Mental Health Surveys found that binge eating disorders were estimated to be 3 to 6 times higher among obese participants (45). Binge-eating disorder (BED) and night-eating syndrome (NES) are two forms of eating disorders that are associated with weight gain, diabetic risk, and metabolic syndrome (46). Binge eating disorders are associated with an earlier onset of overweight and obesity; 30% of those with BED reported childhood obesity (47). Among bariatric surgery patients, the prevalence rate of BED ranges was estimated to reach 47%, which is considered very high (48).

Based on our findings, a large proportion of high-risk participants (72%) was dieting (currently or before) *p* < 0.001. In addition, among all other predictors, dieting contributed to about five times increase in the eating disorders risk (OR = 4.8, CI = 3.38–6.77, *p* < 0.001). Dieting has become a frequent and normalized phenomenon in many cultures, even among young children. Even though dieting may not be the only cause of eating disorders, it is a crucial precursor. According to the National Eating Disorders Association, “normal dieting” may shift into “pathological dieting”; thus, leading to disordered eating risk (49). Besides, the onset of eating disorders had been detected after following diets, especially the restrictive ones (49). Many researchers provide data in this regard. In a study entitled: “Have Our Attempts to Curb Obesity Done More Harm Than Good?”, Memon et al. (14) concluded that dieting, if restrictive, may not help patients lose weight and may cause psychological and physical adverse effects. They also added that dieting induces more risks than benefits as a means to lose weight, exploring the dieting and eating disorders relationship (14). Along with media use, body image dissatisfaction, and weight-related teasing, dieting may contribute to the development of a spectrum of weight-related disorders (50).

Also, a worth noted finding in the current study is that participants had the highest behavioral risk in binge eating (28.9%), followed by excessive exercise (15.6%), rapid loss of body weight (14.1%), pills use (9.9%), and self-induced vomiting (5.9%). Besides, compared to those having a low risk for eating disorders on the EAT-26, high-risk participants significantly reported more disordered behaviors regarding binge eating (34.2 vs. 27.4%, *p* = 0.04), self-induced vomiting (15.6 vs. 3.1%, *p* < 0.001), pills use (22.7 vs. 6.2%, *p* < 0.001), and rapid weight loss (25.8 vs. 10.7%, *p* < 0.001). In a previous cross-sectional study in Italy in 2009, self-induced vomiting was reported by 35.5% of eating disorder patients, with 21.1% having multiple purging with vomiting. Besides, self-induced vomiting was associated with a higher frequency of bulimic episodes, higher levels of depression, longer eating disorder duration, and lower self-determination (51). Eating disorder patients may develop laxative abuse in a mistaken attempt to “feel thin” and “feel empty,” resulting in a variety of serious health complications (52). In some cases, eating disorder patients refuse to re-hydrate, alarming for a lethal health condition, “dehydration” (52). Among Anorexia Nervosa (AN) patients, the core feature predominating is self-starvation resulting in an apparent loss in body weight and fat (53). Anorexia nervosa adolescent females lose more central body fat, while adult females lose more peripheral fat (53). The American Psychiatric Association (APA) claims that the Body Mass Index is typically under 18.5 in an adult individual with anorexia nervosa (1).

### Limitations and strengths

Study findings should be interpreted with caution due to some limitations. Firstly, the cross-sectional design of the study limits reaching a causal inference. Secondly, some data may not be accurate as the questionnaire was self-reported, exploring possible bias in participants' responses. In addition, responders may intentionally under-report eating disorder-related attitudes and behaviors. Besides, we did not perform a clinical diagnosis for the high-risk participants to confirm the results of EAT-26. The conclusions drawn from this study should be treated with caution due to the use of the convenience sampling technique, as it is prone to the challenge of representativeness. However, to our knowledge, this study provides the first data on the prevalence of eating disorder risk among health sciences students and healthcare practitioners in different health professions. Besides, student participants were recruited from multiple universities, pharmacies, clinics, and hospitals across Lebanon, allowing for data generalizability.

## Conclusion and recommendations

This study uncovers an undervalued health-related disorder in Lebanese health science students and healthcare practitioners. Eating disorders have complex and inter-linked etiologies and are beyond having an unhealthy relationship with food. Although it is associated with a high mortality rate in contrast to other chronic diseases, eating disorders are still not valued enough in many societies, especially where stigmatization is common. Public health workers and researchers should promote health and well-being without unintentionally inducing body image dissatisfaction and weight stigma. Eating disorder counseling for health sciences students and healthcare practitioners is recommended, as they are supposed to educate others in their community. Future research may aim to follow up high-risk individuals for clinical diagnosis and treatment initiation. Other correlates with eating disorders may also be examined to direct future interventions on the right path.

## Data availability statement

The original contributions presented in the study are included in the article/supplementary material, further inquiries can be directed to the corresponding author/s.

## Ethics statement

The studies involving human participants were reviewed and approved by ethical approval was obtained from the Ethical Committee at the Lebanese University (#CU-21-18). The patients/participants provided their written informed consent to participate in this study.

## Author contributions

MH: conceptualization, methodology, supervision, and writing—reviewing and editing. HM, GM, NJ, and NY: data curation, writing—original draft preparation, visualization, and investigation. All authors contributed to the article and approved the submitted version.

## Conflict of interest

The authors declare that the research was conducted in the absence of any commercial or financial relationships that could be construed as a potential conflict of interest.

## Publisher's note

All claims expressed in this article are solely those of the authors and do not necessarily represent those of their affiliated organizations, or those of the publisher, the editors and the reviewers. Any product that may be evaluated in this article, or claim that may be made by its manufacturer, is not guaranteed or endorsed by the publisher.
